# Methodological approaches, challenges, and opportunities in the application of Mendelian randomisation to lifecourse epidemiology: A systematic literature review

**DOI:** 10.1007/s10654-023-01032-1

**Published:** 2023-11-08

**Authors:** Grace M. Power, Eleanor Sanderson, Panagiota Pagoni, Abigail Fraser, Tim Morris, Claire Prince, Timothy M. Frayling, Jon Heron, Tom G. Richardson, Rebecca Richmond, Jessica Tyrrell, Nicole Warrington, George Davey Smith, Laura D. Howe, Kate M. Tilling

**Affiliations:** 1 MRC Integrative Epidemiology Unit, University of Bristol, Bristol, UK; 2 Population Health Sciences, Bristol Medical School, University of Bristol, Oakfield House, Oakfield Grove, Bristol, BS8 2BN UK; 3 Institute for Molecular Bioscience, The University of Queensland, Brisbane, Queensland, Australia; 4 Centre for Longitudinal Studies, Social Research Institute, University College London, London, UK; 5 Genetics of Complex Traits, College of Medicine and Health, University of Exeter, Exeter, UK; 6 Frazer Institute, University of Queensland, Woolloongabba, Queensland, Australia; 7 NIHR Bristol Biomedical Research Centre Bristol, University Hospitals Bristol and Weston NHS Foundation Trust, University of Bristol, Bristol, UK

**Keywords:** Lifecourse, Mendelian randomisation, Systematic literature review, Methodology

## Abstract

Diseases diagnosed in adulthood may have antecedents throughout (including prenatal) life. Gaining a better understanding of how exposures at different stages in the lifecourse influence health outcomes is key to elucidating the potential benefits of disease prevention strategies. Mendelian randomisation (MR) is increasingly used to estimate causal effects of exposures across the lifecourse on later life outcomes. This systematic literature review explores MR methods used to perform lifecourse investigations and reviews previous work that has utilised MR to elucidate the effects of factors acting at different stages of the lifecourse. We conducted searches in PubMed, Embase, Medline and MedRXiv databases. Thirteen methodological studies were identified. Four studies focused on the impact of time-varying exposures in the interpretation of “standard” MR techniques, five presented methods for repeat measures of the same exposure, and four described methodological approaches to handling multigenerational exposures. A further 127 studies presented the results of an applied research question. Over half of these estimated effects in a single generation and were largely confined to the exploration of questions regarding body composition. The remaining mostly estimated maternal effects. There is a growing body of research focused on the development and application of MR methods to address lifecourse research questions. The underlying assumptions require careful consideration and the interpretation of results rely on select conditions. Whilst we do not advocate for a particular strategy, we encourage practitioners to make informed decisions on how to approach a research question in this field with a solid understanding of the limitations present and how these may be affected by the research question, modelling approach, instrument selection, and data availability.

**Supplementary Information:**

The online version contains supplementary material available at 10.1007/s10654-023-01032-1.

## Introduction

Diseases diagnosed in adulthood often have antecedents throughout (including prenatal) life [1]. Gaining a better understanding of how exposures at different stages in the lifecourse influence health outcomes is key to elucidating the potential benefits of specific disease prevention strategies.

A lifecourse approach recognises the contribution of long-term biological, behavioural, and psychosocial processes that operate across an individual’s lifecourse, or across generations [2, 3]. Kuh et al. previously defined lifecourse epidemiology as the study of physical or social exposures during gestation, childhood, adolescence, earlier adulthood and later adult life on later health or disease risk [2]. In practice, operationalising this can be complex; by definition, exposures should precede outcomes, and so almost any study of an exposure in relation to an adult health outcome could arguably be considered a lifecourse study. Here, our focus is on methodological issues pertinent to the application of Mendelian randomisation (MR) to lifecourse studies; these issues are relevant where there is a large time gap between exposures and outcomes. Therefore, we consider the following types of study as falling within lifecourse epidemiology: (1) the effects of pre-gestation, gestation, early life, childhood, or adolescent exposures on adult outcomes; (2) the effects of adult exposures on adult outcomes when the adult exposure is related to a particular stage/phase of adulthood, such as menopause (e.g. the effects of age at menopause on cardiovascular disease), (3) the effects of repeated measures of a time-varying exposure on a later outcome.

Whilst a lifecourse approach provides a persuasive framework for conducting epidemiological research, mediation (and the effect of this on the interpretation of total effects), time-varying confounding (when confounders have values that change over time) and intermediate confounding (a confounder of the mediator-outcome relationship) are highly likely in studies with earlier life and time-varying exposures and later life health outcomes [4, 5]. Intergenerational and family level factors may also contribute to further distinctive sources of confounding in multigenerational studies. Approaches to interrogate causality by minimising confounding are therefore of importance to strengthen causal inference in a lifecourse setting [6, 7].

MR exploits the random assortment of genetic variants, independent of other traits, to enable analyses that largely mitigate against distortions resulting from confounding and reverse causality [8]. This is a key motivation behind using a MR approach, which estimates the causal effect of modifiable risk factors under three assumptions; the instrumental variables used must (1) be associated with the exposure of interest (‘relevance’), (2) not share common causes with the outcome (‘independence’ or ‘exchangeability’) and (3) not affect the outcome other than through the exposure (‘exclusion’). Several statistical methods have been proposed for MR with individual-level as well as summarised data. In a one-sample setting with individual-level data, a causal effect estimate is often obtained using the two-stage least-squares (2SLS) method [9]. It is more common for two-sample investigations to use summarised data. In addition, at the introduction of MR, it was recognised that the association of genetic variants with exposures could change with age, which needed to be considered in interpretation [10, 11].

The application of MR to lifecourse research questions has two key challenges. Firstly, we are interested in isolating the causal effects of age-specific exposures. MR studies typically use a single measurement of an exposure to estimate its effects on an outcome (henceforth termed “standard” MR) and genes are invariable across the lifecourse. As such, results obtained are often interpreted as the average lifetime effect of the genetically predicted exposure, or genetic liability for an exposure if that exposure is binary [12]. Whilst this approach is sufficient for some exposures, it requires extension to address lifecourse questions. This extension is possible in cases where inherited genetic variants have different effects at different time points in the lifecourse (within a population), allowing us to separate time-varying effects of certain exposures [13–15]. Secondly, some lifecourse research questions involve the exploration of parental exposures. The inclusion of multiple generations brings additional analytical and methodological challenges due to common confounding and genetic relatedness.

This systematic literature review has two core aims. Firstly, to identify MR methods that have been developed to evaluate or conduct lifecourse epidemiological investigations and secondly, to systematically review previous work that has utilised MR to elucidate the impacts of risk factors from different stages of the lifecourse on later life outcomes. These studies fulfil the criteria outlined in the STROBE-MR guidelines, and specifically to the criterion of whether effect estimates previously derived would generalise to other exposure periods [16, 17].

## Methods

### Search strategy and eligibility criteria

The protocol for this systematic literature review was registered in the International Prospective Register of Systematic Reviews (PROSPERO) as CRD42022314287 and was conducted in line with the 2020 Preferred Reporting Items for Systematic Reviews and Meta-Analyses (PRISMA) guidelines [18]. We searched for lifecourse epidemiology studies, defined as: (1) the effects of pre-gestation, gestation, early life, childhood, or adolescent exposures on adult outcomes; (2) the effects of adult exposures on adult outcomes when the adult exposure is related to a particular stage/phase of adulthood, such as menopause (e.g. the effects of age at menopause on cardiovascular disease), (3) the effects of repeated measures of a time-varying exposure on a later outcome. (See Supplementary file 1) [19]. Studies were eligible from any geographical location, with individuals from any age group and which included a MR study design (i.e., a study using genetic variants to determine whether there is a causal relationship between a modifiable risk factor and an outcome). We include as an “MR study” any study that uses genetic variants related to an exposure of interest to understand the causal nature of the relationship between that exposure and an outcome of interest. This includes studies where the genetic variants are used as an instrumental variable, and those where the association between the genetic variants and the outcome under study is analysed outside of an instrumental variable framework. Searches included any papers published prior to 12 June 2023 in MEDLINE (PubMed), Embase (Ovid), Medline (Ovid) and MedRXiv. The search and full-text review were restricted to articles published in English. Outcome measures were any measure of health status or disease from a life stage after the exposure was measured. Study designs that do not use MR methods were not appraised. Treatment guidelines documents were excluded (Supplementary file 2).

### Data extraction and analysis

Within the final list of papers, we separated methodological manuscripts that presented or tested an approach to lifecourse MR from applied papers that only presented the results of a specific lifecourse analysis. For methodological manuscripts that presented or tested an approach to lifecourse MR we recorded: author, baseline year of data collection, aim, methodological approach, challenges in methodological application, simulation scenarios, sample size, and assumptions. When an applied element was included in the manuscript, we also recorded: exposure, exposure age(s) in years, outcome and outcome age(s) in years. We extracted the following from applied studies that presented the results of a specific lifecourse analysis: author, baseline year of data collection, aim, exposure, exposure age(s) in years, outcome and outcome age(s) in years. Title and abstract and then full-text screening was conducted in duplicate by two investigators (G.M.P and P.P.) and extraction in duplicate by two investigators (G.M.P and C.P.). Discrepancies were resolved by consensus. A narrative synthesis was performed. The evaluation of study quality by conducting a bias assessment was not considered relevant here, since we were not collating evidence to answer one applied question [20, 21].

## Results

**Fig. 1:**
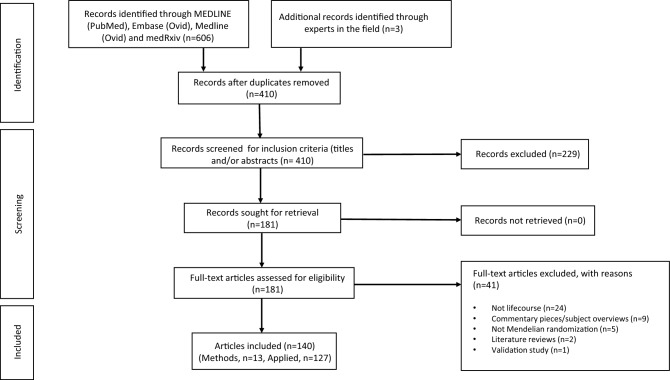
PRISMA flow chart illustrating selection of studies. PRISMA, Preferred Reporting Items for Systematic Reviews and Meta-Analyses

Our search generated 407 records. Three additional records were identified through conversations with experts in the field. After screening titles and abstracts, 181 manuscripts were assessed for eligibility. Of these, 140 articles were deemed eligible for inclusion in this systematic review (Fig. 1). Thirteen studies presented or tested an approach to lifecourse MR [12–15, 22–30] and 127 presented the results of a specific lifecourse analysis without an emphasis on exploring or explaining a methodological approach [31–157]. If a study fit the criteria for the former section, it was not included in the latter.

### Studies presenting or testing an approach to lifecourse MR

Of the 13 studies presenting and/or testing approaches to lifecourse MR, four focused on the impact of time-varying exposures on the interpretations of “standard” MR techniques [12, 23, 26, 27]. These additionally outline methods to assess and/or lessen potential bias. Five presented methods for analysing repeat measures of the same exposure. These comprised functional principal component (FPC) analysis through conditional expectation (PACE) followed by a two-stage functional residual inclusion (2SFRI) inverse variance weighted multivariable MR (IVW-MVMR), g-estimation of structural nested cumulative failure models (SNCFTMs) and g-estimation of structural mean models (SMM) [13, 15, 22, 25, 28]. Our definition of lifecourse studies, which includes the effects of repeated measures of the same time-varying exposure on a later outcome, connects lifecourse MR to g-estimation, which has been applied in several studies to adjust for time-varying confounding in traditional epidemiological settings [158, 159]. In addition, four studies described novel methods that have been developed for intergenerational studies investigating a parental or grandparental exposure whilst the outcome of interest is assessed in offspring. These have used structural equation models (SEM) or the statistically equivalent weighted linear model (WLM), as well as one-sample GRS analysis and gene-by-environment (G × E) MR [14, 24, 29, 30].

#### Implications of time-varying exposures for the interpretation of “standard” MR

There are potential limitations regarding the use of “standard” MR techniques to interpret relationships between an outcome and an exposure that change over the lifecourses. D’Urso et al. highlight issues when using MR to assess the validity of hypotheses relating to the Developmental Origins of Health and Disease (DOHaD), such as the Barker hypothesis, which proposes that the origins of chronic diseases of adult life lie in foetal responses to the intrauterine environment [26]. “Standard” MR methods do not take into account the relationship between maternal and offspring genotypes and, as a result, may produce inflated type 1 error rates. Standard errors may be too small in the presence of cryptic relatedness due to there being less genetic variation in the sample. A conditional analysis framework is recommended using an unweighted or weighted maternal allele score corrected for offspring genotypes [26].

Results from “standard” MR techniques are often interpreted as average lifetime effects of the exposure, i.e., the cumulative effect of the exposure level from conception and through the lifecourse. Labrecque et al. propose an alternative interpretation for exposures that vary over time. They suggest the effect should be interpreted using a counterfactual framework approach, shifting the entire exposure trajectory by one unit of time *k* (a timepoint of observation, where *k* = 0 at conception) [23]. Labrecque et al. argue that different effects would be estimated at different exposure time points if the relationship between the genetic variants and the exposure changes over time. Thus, a “standard” MR approach may produce biased results. They initially provided an empirical example to estimate the lifetime effect of body mass index (BMI) on systolic blood pressure using the rs9939609 variant. They then simulated a longitudinal relationship to estimate BMI as an exposure at age 30 and 50 years and concluded that when the genetic variable-exposure relationship was constant over time, estimates were unbiased with respect to the lifetime effect at both ages. In all other scenarios, however, they show the estimate differed, and this bias was sensitive to the strength of relationship between the genetic variant and exposure as well as the timing of measurement of both exposure window and outcome.

Previous studies have explored whether age modifies the relationship between the genetic variants and exposure [10], however, investigations are limited. Most studies that have addressed this have investigated body composition, BMI or other measures of body size. To assess how time-varying genetic effects may impact MR effect estimates, Labrecque et al. and others suggest looking at a statistical interaction between the genetic variant and age in relation to the exposure [13, 106, 107, 112, 115, 160]. Following this, Labracque et al. propose plotting the relationship between the genetic instrument and the exposure stratified by age in samples with sufficient variation in age. They additionally show that patterns in age-varying genetic relationships may be exposure specific [27]. This has been shown in applied studies [10, 13, 106, 107, 112, 115, 160].

Morris et al. clarify the causal estimates that are estimated by MR when applied to a single measure of a time-varying exposure with time-varying genetic effects [12]. They consider a situation where there is one genetic instrument, a time-varying continuous exposure assessed on two occasions, and a single measure of an outcome. They also note the genetic instrument cannot affect the exposure measured at different occasions in isolation. Instead, they argue that the instrument underlies all possible exposure measurements across the lifecourse through a genetic liability, so a change in genotype changes both measures of the exposure. Simulations demonstrate that the Wald Ratio MR estimator recovers the correct causal effect in all scenarios assessed, even where time-varying genetic associations were present. Morris et al. showed that MR estimates differ between measurements of time-varying exposures because MR is estimating the total effect of the exposure trajectory on the outcome rather than the effect of the exposure at a specific point in time. Further details of each of these approaches can be found in Supplementary file 3.

#### Methodological approaches to analysing repeat measures of the same exposure over the lifecourse in an MR framework

MR methods proposed to estimate the effects of repeat measures of the same exposure across the lifecourse have been developed in response to the concern that a single measurement of a time-varying exposure may not be adequate in capturing all time-varying information: a single measure of a time-varying exposure could underestimate the relationship between the exposure variable and the outcome variable due to the failure to capture long-term change [161]. Importantly, in this context, later stages of lifecourse exposures often depend on the earlier stages of the same exposure, whilst the reverse is not true.

Cao et al. developed two methods to combine functional data analysis (to describe the trajectory of the exposure) with MR, to test the causal effect of a time-varying exposure on a binary outcome [22]. They use functional principal component (FPC) analysis through conditional expectation (PACE) to model the exposure trajectories, and then test whether a summary measure of the trajectory is related to the outcome using the two-stage residual inclusion (2SRI) approach. Their methods examine the evidence against the null hypothesis of no causal effect, but do not estimate the causal effect. The first method (PACE + 2SRI) assumes that the time-varying exposure variable has a cumulative effect on the risk of disease, and that the genetic effects on the exposure do not vary over time. The cumulative value of the exposure between two timepoints can be obtained by integration. The first stage obtains the residuals from regressing this cumulative exposure on the instrument (and any non-time-varying covariates). The second stage then relates these residuals to the outcome via a logistic regression model. For the second method (PACE + 2SFRI), they allow a time-varying genetic effect on the exposure variable but assume that the effect of the exposure and the fitted residual on the outcome are constant over time. In this case, the first stage is a functional linear model for the time-varying exposure, and the second stage relates the outcome to the fitted residuals and to the detrended exposure (functional residual inclusion). The authors showed that this method outperformed “standard” MR analysis with a single measurement at one time point, with higher statistical power in simulation studies using the functional data analysis-based methods, even when the disease outcome was simulated to depend not on the cumulative exposure, but on the first three functional principal component scores from PACE.

**Fig. 2:**
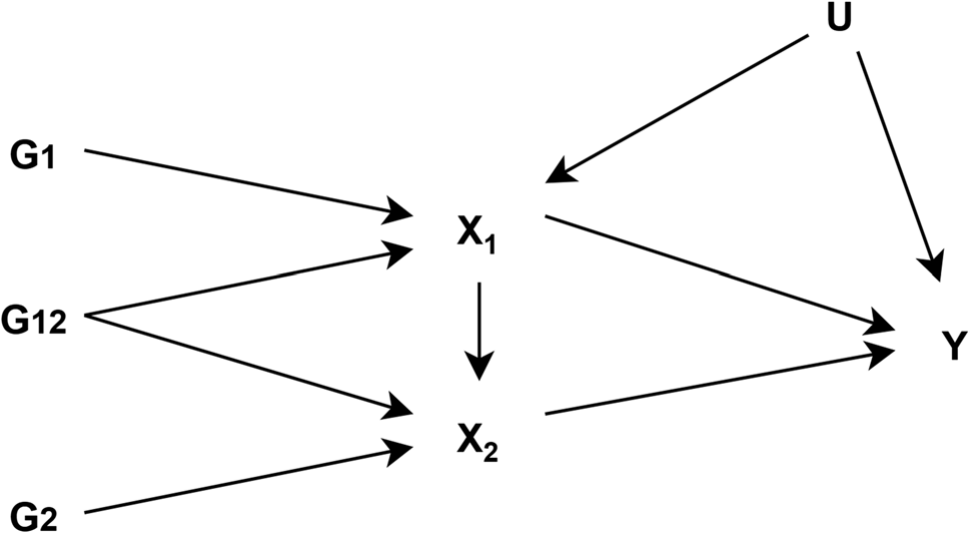
Latent exposure model with two periods of exposure (adapted from Sanderson et al. [163]). *G*_*1*_ is a set of genetic variants associated with the earlier exposure (*X*_*1*_) *G*_*2*_ is a set of genetic variants associated with the later exposure (*X*_*2*_), *G*_*12*_ is a set of genetic variants associated with both *X*_*1*_ and *X*_*2*_

Another method employed to assess repeat measures of the same exposure over the lifecourse is inverse variance weighted multivariable MR (IVW-MVMR) [13, 15]. IVW-MVMR can be used to estimate the independent direct effects of several highly correlated exposures on an outcome, conditional on all the other exposures included in the model. It is useful in the context of mediation analysis [162], to estimate the effects of several repeated measures of the same exposure, or to isolate the effects of related phenotypes. Sanderson et al. explore the use of IVW-MVMR to estimate the direct effect of a single exposure at different time points in an individual’s lifetime on an outcome (Fig. 2) [15]. For multiple measurements to be included in a IVW-MVMR the genetic variants must have different effects on each exposure included in the model and these effects must not be a linear function of the others. The interpretation of the estimate is the effect of having a liability associated with a unit higher level of exposure at one occasion while keeping the liability for exposure at a separate occasion constant. Richardson et al. applied this approach to evaluate whether body size in early life has an independent effect on risk of disease in later life, or whether the effect seen is a result of body size in childhood being mediated by body size in adulthood [13]. They use univariable MR to estimate total effects of early body size, and IVW-MVMR to estimate direct effects of early and adult body size. This approach suggests univariable analyses cannot identify critical or sensitive periods of exposure but can detect an effect of a difference in the cumulative lifetime exposure, which is a notion critiqued by Labrecque et al., highlighted earlier in this review [23, 27]. If measures of the exposure at different time periods are available, and genetic instruments capable of reliably separating time-varying effects exist, it is possible to identify whether the exposure effects are stable over time or whether sensitive/critical periods exist in the lifecourse using IVW-MVMR. In theory the more time periods we have should allow more granular inference into critical windows. However, whilst this method can narrow down or exclude periods, it cannot strictly identify important periods if the genetic effects on the periods included are correlated with genetic effects on excluded periods.

Further attention has been bought to the importance of mitigating misspecification when running IVW-MVMR to estimate the effects of a single exposure during distinct time periods [164]. Tian and Burgess caution that this may otherwise result in the model’s poor performance with estimates suffering from unpredictable bias in both magnitude and direction [164, 165]. Correctly specifying when the outcome is a discrete function of the exposure at the precise time points at which the exposure was measured is therefore key. To run IVW-MVMR to answer lifecourse questions, Tian and Burgess argue that it is essential the exposure periods estimated represent distinct periods in the lifecourse where effects on the outcome are limited to a particular time period. This underlines ongoing methodological debates in this field. Sanderson et al. argue that any effect through a time period excluded from the model will form part of the effect estimated, asserting that, that effect can still be interpreted as the causal effect. Whilst being able to separate the genetic instruments for each period is important, running analyses on genetically predicted effects in small age-bands will almost certainly result in weak instruments and yield biased results.

**Fig. 3:**
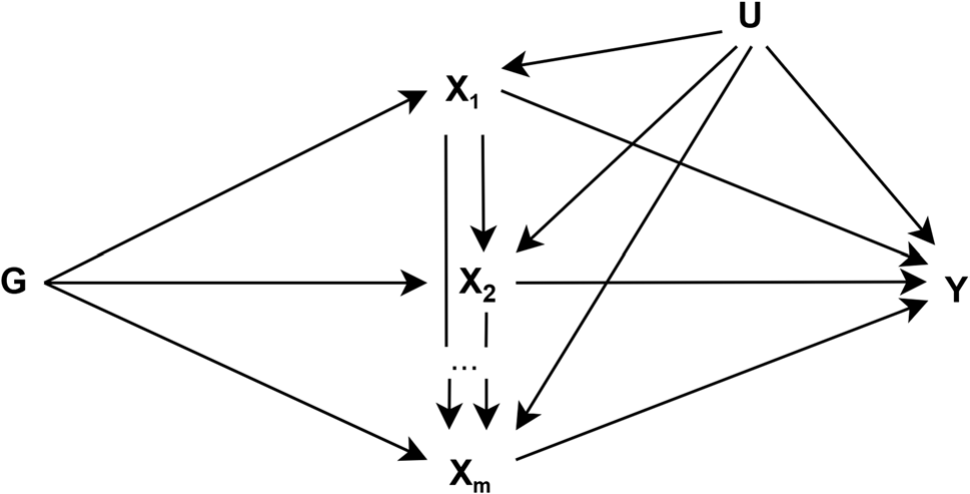
Causal diagram for instrumental variable analyses representing a scenario with a time-varying exposure (adapted from Shi et al. 2022 [28]). *G* indicates a set of genetic variants each associated with at least one of the exposures (*X*_*m*−*p*_,…, *X*_*m*–*1*_, *X*_*m*_)

The application of g-estimation of structural nested cumulative failure models (SNCFTMs) and g-estimation of structural mean models (SMM) was proposed by Shi et al. for the estimation of MR models with a time-varying exposure (Fig. 3) [25, 28]. The interpretation of results from estimation for these models depends on the availability of data for the time-varying exposure. SNCFTMs can be used to estimate the causal effect of a time-varying treatment on a failure time outcome under the assumption that all time-varying confounders have been measured and that failure is rare under all possible treatment values [166]. Shi et al. describe an adaptation of this use of SNCFTMs, incorporating IV-type assumptions [25]. Whilst confirmation of the validity of the method was achieved via simulations, analyses indicated that MR with time-varying treatments and failure time outcomes using SNCFTMs require large sample sizes (*n* = 10,000; *n* = 25,000 or *n* = 50,000). In addition, authors note that this method should only be used with rare outcomes. In the application of g-estimation of SMMs to MR analyses, Shi et al. consider three types of causal effects that can be targeted when the exposure is time-varying: the effect of exposure at a single time point on the outcome (point effect), the effect of exposure during a period on the outcome (period effect), and the effect of exposure throughout the lifetime on the outcome (lifetime effect) [28]. This approach highlighted two key challenges in estimating and interpreting period effects from MR analyses. The first is defining the period of interest. The second is the choice of time scale (e.g., time since conception or time since enrolment). In the context of additive causal effects for continuous outcomes, the authors note that g-estimation of SMMs and two-stage least squares (2SLS) MR yield similar estimates. SMMs can be naturally extended to many settings, including accommodating binary and failure-time outcomes and estimating effects on the multiplicative scale. SMMs are also semiparametric, and therefore avoid some of the parametric assumptions of 2SLS. Further details on these methodological approaches discussed along with their limitations are presented in Supplementary file 3.

The methodological assumptions underlying the methods we present here vary greatly and require thorough consideration prior to running analyses. On top of this, very careful consideration is required for instrument selection when applying MR to lifecourse research questions. We therefore do not advocate for a particular strategy but encourage practitioners to think through their research question, instrumental variables, and data availability in-depth before pursuing a particular MR approach within a lifecourse setting. Table 1 comprises key considerations for analysts that are thinking about conducting a lifecourse investigation using MR techniques.

**Table 1:** Key methodological considerations when implementing a Mendelian randomization approach to conduct lifecourse research

Method	Phenotypic data required		SNP set required	Adjustments (if any)	Data level required	Generation (one or two)	MR effect	MR estimation	Estimate	Interpretation	Methodological assumptions
	Exposure data	Outcome data									
G-estimation of SMM	Measure of phenotype from specific timepoint	Measure of phenotype from a life stage after exposure data	SNPs only associated with exposure phenotype from specific timepoint and no other timepoints (association threshold may vary)	–	Individual	One	Period	Univariable MR	–	The effect of the phenotype of interest from a specific timepoint independent of pathways comprising considered phenotype measures from other timeframes in the lifecourse	Genetic variants strongly associated with the phenotype of interest from a specific timepoint do not have a pathway effect through another time period
G-estimation of SMM; IVW-MR; 2SLS-MR	Measure of phenotype from specific timepoint.	Measure of phenotype from a life stage after exposure data	Full set of SNPs associated with exposure phenotype from specific timepoint (association threshold may vary)	–	Individual; summary	One	Lifetime	Univariable MR	Total	The effect of the phenotype of interest from a specific timepoint including pathways comprising considered phenotype measures from different timeframes in the lifecourse	Genetic variants strongly associated with the phenotype of interest from a specific timepoint do not have a pathway effect through another time period
G-estimation of SMM; IVW-MVMR; 2SLS-MVMR	Measure of phenotype from specific timepoint.	Measure of phenotype from a life stage after exposure data.	Full set of SNPs associated with exposure phenotype from specific timepoint (association threshold may vary), plus full set of SNPs associated with alternative timepoint(s)	Control for measure of same phenotype as exposure from different timepoint(s)	Individual; summary	One	Period	Multivariable MR	Direct	The effect of the phenotype of interest from a specific timepoint including pathways comprising considered phenotype measures from different timeframes in the lifecourse, conditioning on phenotype measures from other time periods	There is variation in the genetic variant phenotype association across the time periods included in the estimation
G-estimation of SNCFTM	Repeat longitudinal measurements of phenotype between two time points.	Measure of phenotype from a life stage after exposure data.	Full set of SNPs associated with exposure phenotype (association threshold may vary)	–	Individual	One	Period or lifetime	–	Cumulative	The marginal cumulative risk under nondynamic treatment strategies across a period	Genetic variants strongly associated with the phenotype of interest from a specific timepoint do not have a pathway effect through another time period
PACE + 2SFRI	Repeat longitudinal measurements of phenotype between two time points	Measure of phenotype from a life stage after exposure data	Full set of SNPs associated with exposure phenotype (association threshold may vary)	–	Individual	One	Period or lifetime	–	Cumulative	The cumulative effect of the phenotype of interest across a period or the lifecourse	Genetic variants have time-varying effects on the exposure variable and the exposure variable has a cumulative effect on the disease risk
Genomic SEM or WLM (yielding a good approximation to the SEM) to conduct two-sample MR	Measure of phenotype in general population (i.e. does not specifically need to be in parents or offspring)	Measure of parental and offspring phenotype (these can come from separate samples)	Maternal (paternal) and offspring SNPs that are associated with maternal (paternal) exposure phenotype	Condition on parental or offspring genotype	Summary	Two	Parental	Univariable MR	Direct (offspring) + indirect (parental)	The causal effect of an intrauterine phenotype (separating direct offspring genotype effects from indirect maternal/paternal genotype effects) on an offspring outcome	Partitioning the outcome into maternal (paternal) and offspring specific genetic effects allows for the estimation of both parental and offspring causal effects on offspring outcomes
One-sample GRS analysis	Measure of parental/offspring phenotype	Measure of offspring phenotype	Maternal (paternal) and offspring SNPs that are associated with maternal (paternal) exposure phenotype	Adjust for offspring genotype (and other parent if relevant)	Individual	Two	Parental	Univariable MR	Direct (offspring) + indirect (parental) parental	The causal effect of an intrauterine phenotype (separating direct offspring genotype effects from indirect maternal/paternal genotype effects) on an offspring outcome	Partitioning the outcome into maternal (paternal) and offspring specific genetic effects allows for the estimation of both parental and offspring causal effects on offspring outcomes
G × E MR	Offspring measure on parental phenotype as proxy for parental phenotype	Measure of phenotype from a life stage after exposure data	Offspring SNPs associated with exposure phenotype as proxy for parental genetic data	–	Individual	Two	Parental	Univariable MR	–	The causal effect of an exposure in pregnancy when maternal genotype is unavailable	Offspring genotype is a suitable proxy for maternal genotype

2SFRI = two-stage functional residual inclusion; 2SLS = Two-stage least-squares regression; G x E = gene-by-environment; GRS = genetic risk score; IVW = Inverse variance weighted; MR = Mendelian randomisation; MV = Multivariable; PACE = principal component analysis through conditional expectation; SEM = structural equation model; SMM = Structural mean model; SNCFTM = structural nested cumulative failure model; SNP = Single nucleotide polymorphisms; WLM = weighted linear model

#### Novel methodological approaches to handling parental exposures in relation to offspring outcomes

**Fig. 4:**
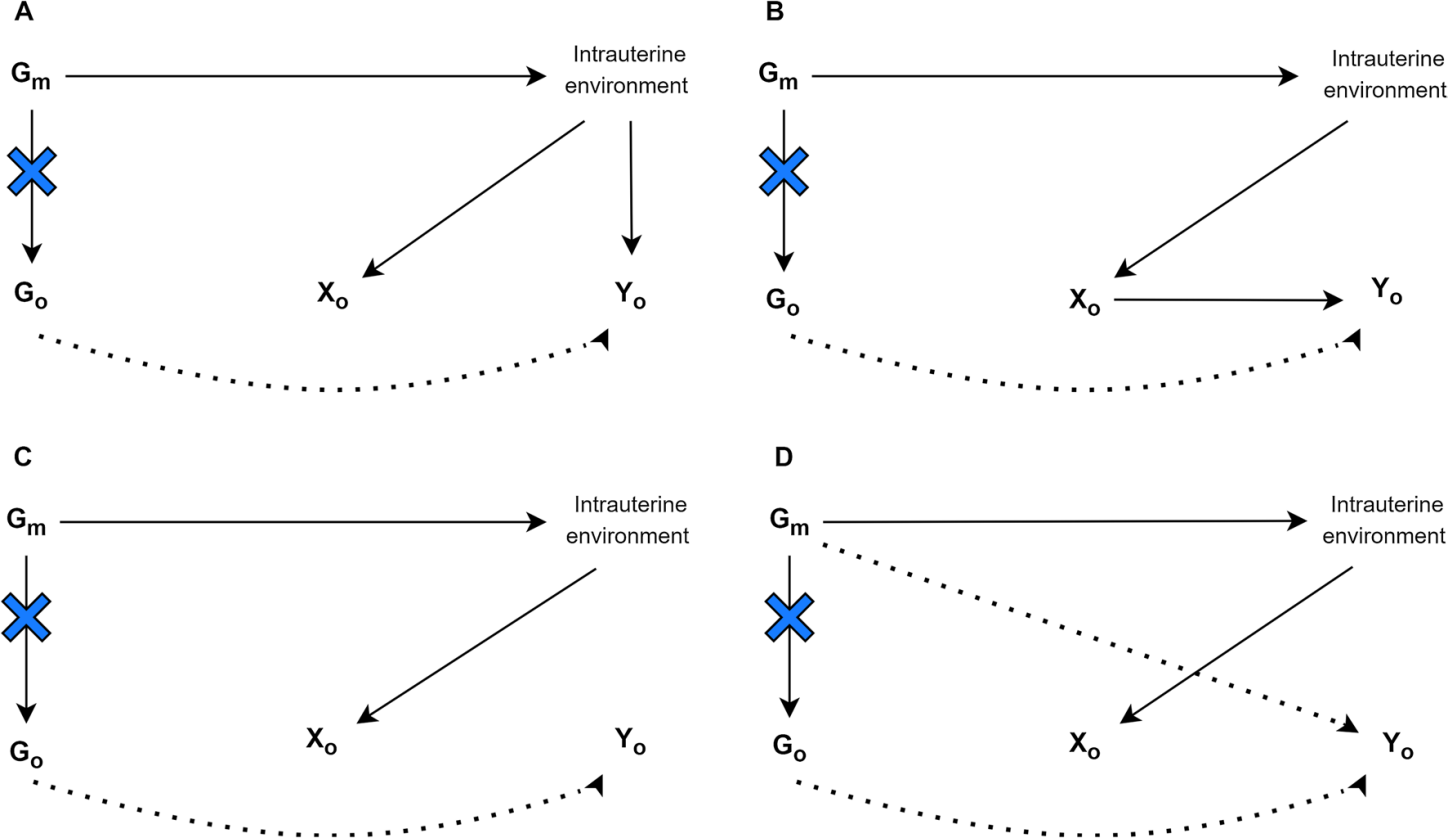
Four credible ways in which maternal genetic variants can be related to an offspring exposure (*X*_*O*_) and offspring outcome (*Y*_*O*_). *G*_*m*_ is a set of maternal genetic variants *G*_*O*_ is a set of offspring genetic variants. Blue crosses indicate the act of conditioning on maternal or offspring genotype, blocking the association between maternal and offspring variables. Dotted paths show paths in which the maternal genotype can be related to offspring phenotype that are not to do with the intrauterine environment (adapted from Evans et al. [29] Moen et al. [95] and Warrington et al. [14])

Novel methods have been developed for intergenerational studies investigating a parental or grandparental exposure whilst the outcome of interest is assessed in offspring. All of the studies we identified in this section relate maternal genotypes to offspring outcomes and establish the causal effect of a maternal exposure, e.g., smoking during pregnancy, on offspring health. Yang et al. used a proxy gene-by-environment (G × E) MR approach to explore maternal effects on offspring phenotypes where maternal genetic information was unavailable [30]. They validated this approach by replicating a known effect of maternal smoking heaviness on offspring birthweight using the rs16969968 variant in *CHRNA5*. They then applied it to explore effects of maternal smoking heaviness on offspring later life outcomes and on birthweight of participant’s children. Yang et al. demonstrated how G × E MR can be used to test transgenerational causal effects. Further studies included in this section emphasise the need to condition on offspring genotype to avoid including its effect on the outcome of interest. Earlier non-MR human genetic association studies have estimated maternal genetic effects on offspring phenotypes through conditional genetic association analysis of genotyped mother–offspring pairs [167]. This separation of genetic effects into maternal and offspring components is important as maternal and offspring genotypes are correlated. Consequently, any association between maternal genotype and offspring outcome may be mediated by offspring genotype (Fig. 4) [14, 29]. Thus, as described above, naïve two-sample MR approaches in unrelated sets of individuals without accounting for the correlation between maternal and offspring genotype effects may result in erroneous conclusions regarding causality.

Two MR approaches, described by Warrington et al. and Evans et al. use structural equation modelling (SEM) [166] to account for the correlation between maternal and offspring genotypes [14, 29]. Evans et al. developed a statistical model that can be used to estimate the effect of maternal genotypes on offspring outcomes, conditional on offspring genotype using both individual-level and summary data. The authors demonstrate this approach using the following example: birthweight of the individual, birthweight of the individual offspring, and the mother’s own genotype (SNP). The genotypes of the individual’s mother (their offspring’s grandmother) and the genotype of the individual’s offspring are considered latent unobserved variables. The causal path between the individual’s own genotype and both their mother and offspring’s latent genotype is set to 0.5, according to quantitative genetics theory. The estimated maternal and offspring effects on the observed phenotype, which refer to maternal and offspring genetic effects on birthweight, are also estimated. The resulting maternal and offspring genetic effects can subsequently be combined with SNP-exposure estimates for the maternal exposures that the investigator is interested in, in a two-sample MR framework.

Warrington et al. ran GWAS of own offspring genetic variants in relation to birthweight, and maternal genetic variants in relation to their offspring’s birthweight. They then partitioned the lead SNPs, representing independent association signals, into categories based on maternal and/or offspring genetic contributions to birth weight. To achieve this, they use the same SEM [166] as described in Evans et al. [29] to account for the correlation between offspring and maternal genotypes to provide unbiased estimates of maternal and offspring genetic effects on birthweight. This method gives an indication as to which genetic associations are driven by the maternal and which by the offspring genomes. To extend the estimates of adjusted maternal and foetal effects genome wide, the authors developed a weighted linear model (WLM) which yields a good approximation of the SEM but is less computationally intensive. They used WLM-adjusted estimates in downstream analyses to identify maternal and offspring specific mechanisms that regulate birthweight and to investigate genetic links between maternal traits and birthweight. The authors applied two-sample MR to estimate causal effects of intrauterine exposures on offspring birthweight. Authors selected SNPs associated with each exposure and regressed the WLM-adjusted maternal effects on birthweight for those SNPs against the effect estimates for the maternal exposure, weighting by the inverse of the variance of the maternal exposure effect estimates. Similarly, the authors used WLM-adjusted offspring effects to estimate the causal effect of the offspring’s genetic potential on their own birthweight and compare the results with the estimated maternal causal effects.

Moen et al. investigate whether a genetic risk score (GRS) of maternal SNPs associated with offspring birthweight is also associated with offspring cardiometabolic risk factors, after controlling for offspring GRS using a one-sample GRS analysis approach. They use a large dataset and perform primary analyses testing the relationship between maternal GRS and each of the offspring risk factors, whilst conditioning on the offspring GRS. They also explore father-offspring pairs to investigate whether there is evidence for a postnatal environmental effect (genetic nurture or dynastic effects) rather than an intrauterine environmental effect. In executing these analyses, the authors employ a LMM which accounts for the non-independence between siblings. They modelled the maternal (paternal) GRS, offspring GRS, age, sex and measurement occasion. The non-independence between siblings and relatedness between parents and offspring was modelled using a genetic relatedness matrix in the random effects part of the model [24]. Importantly, a one-sample GRS analysis can also be used in single generational setting. Further detail on applied results, assumptions and limitations for these methods are provided in Supplementary file 3. It may be helpful to consider some of the key aspects and requirements for running a multigenerational lifecourse MR analysis, presented in Table 1.

### Applied MR studies presenting results of a lifecourse analysis

Of the 127 studies applying lifecourse MR methods, included in this review, 51% (65/127) estimated effects in just one generation, 42% (53/127) looked at intergenerational effects and 7% (9/127) estimating both. Of the one (and one and two) generational studies employed in this review, 51% (38/74) estimated the effect of exposures at birth, birth to/and childhood, birth to/and adolescence or birth to/and adulthood, 35% (26/74) at childhood, childhood to/and adolescence or childhood to/and adulthood, and 14% (10/74) at adolescence or adulthood. Within those focused on single generational effects, 42% (27/65) looked at birth weight, 38% (25/65) comprised other body composition measures, including adiposity traits, BMI, body size, obesity, waist-to-hip ratio, and body fat percent. Single generation studies additionally included estimating the genetically predicted effects of age at menarche, pubertal age (timing), first sexual intercourse, sleep duration, offspring fasting glucose and type 2 diabetes, genetic liability to juvenile idiopathic arthritis, disordered eating pattern, alcohol consumption and DNA methylation at the HLA locus. Amongst the studies that estimated intergenerational effects, 28% (15/53) examined body composition as exposure measures. These included maternal and paternal BMI as well as maternal adiposity, central obesity, and height. Other exposures examined in an intergenerational setting are included in Supplementary file 4. All of the two-generational studies estimated effects of maternal exposures, with two studies also examining paternal exposures [64, 73]. Outcomes addressed in the studies incorporated in this review are varied and can be found in Supplementary file 4.

## Discussion

In this systematic literature review, we extracted and summarised findings from studies presenting and/or testing approaches to lifecourse MR as well as those presenting results of a specific lifecourse analysis. Among the former, we focused on papers addressing time-varying or lifecourse processes through interpretations of results from “standard” MR techniques. “Standard” MR techniques have focused on estimating lifetime effects of an exposure, i.e., the cumulative effect of the exposure level from conception and through the lifecourse. Labrecque et al. propose that MR estimates of the same exposure assessed at different ages vary in the presence of time-varying genotype-exposure associations, and this represents bias in estimates of a lifetime causal effect. In response, Morris et al. proposed that “standard” MR is not estimating the causal effect of an exposure as it manifests at a given time period, but the causal effect of the underlying exposure liability. Thus, a hypothetical change in genotype would affect all manifestations of the exposure.

In addition, we summarised papers employing a methodological approach for repeat measures of the same exposure over the lifecourse. The methods described here enhance capability for causal inference of lifecourse effects, however, there are clear limitations. One method comprised the FPC analysis through PACE, with the limitation that this method was developed for hypothesis testing, not for estimation of causal effects [22]. Another technique was IVW-MVMR, which can separate influences across the lifecourse under some but not all causal scenarios. Estimates used are based solely on body size and BMI data from the UK Biobank [168, 169]. These findings should be evaluated in more cohorts when sample sizes make this possible. This is particularly important as it has been shown that UK Biobank participants are highly selected, which can be problematic for instrumental variable analyses [168, 168]. In addition, a g-estimation of SNCFTMs was explored. If the rare failure assumption does not hold, however, estimates from this approach may be invalid. Informative MR analyses will additionally require sample sizes much larger than those presented. A g-estimation of SMM was also described. Due to wide variations in age at first visit and short duration of follow-up in the data used, authors were limited to using time since enrolment in the study as the time scale, which implies the added assumption that the period effect is homogeneous across age. The plausibility of this assumption is not only specific to the exposure–outcome relationship of interest, but also depends on the variability in age.

Papers comprising methodological approaches for intergenerational effects or pregnancy/birth exposures emphasised the importance of a statistical model that can estimate the effect of maternal genotypes on offspring outcomes, conditional on offspring genotype. On a related note, carrying out MR of own birthweight using only genetic variants of the individual is likely to result in inaccuracies. This is because foetal growth and subsequently birthweight may be influenced by both foetal and correlated maternal genotypes [72].

As a further test of model assumptions, negative controls may be employed when applying MR to lifecourse epidemiology. For example, in the investigation of repeat measures of the same exposure over the lifecourse, testing a negative control outcome by estimating the direct effect of an exposure in adulthood on an outcome at an earlier life stage will help to decipher whether results being generated are reliable [115].

Additional methodological studies have addressed the importance of gene–gene and gene-environment interactions in shaping the genetic architecture of certain phenotypes [170]. Whilst the current methods presented in our review are not suitable to address this research area, this provides an interesting area for future developments.

The aforementioned MR methods rely on genome-wide association studies (GWASs). Several GWASs are usually meta-analysed to increase power using a fixed-effect approach, which assumes a common true genetic effect across studies. Random-effects models are also employed, though have limited power in comparison. It has been observed that if the genetic effects change with age both fixed-effect or random-effects meta-analysis produce biased estimates of the combined genetic effect [171]. Since the MR methods presented in our review assume that genetic effects may vary with age, one option is to run GWASs on specific age categories and, if possible, apply meta-analysis in each age category. This is an approach most frequently taken in the studies presenting results of specific lifecourse analyses highlighted in this review. Alternatively, meta-regression may be used to relate between-study heterogeneity to age and estimate both main and age-varying genetic effects [171]. These data may then be applied within a MR framework.

Among the studies presenting results of specific lifecourse analyses, data availability limitations were apparent. Studies focusing on one generational research are largely confined to the exploration of questions regarding body composition, since these have the strongest instrumental variables. In addition, these data are often more commonly available on a large scale in most longitudinal cohorts. This emphasises the need for pooling data across studies to maximise power, highlighting the value of a Lifecourse MR consortium, which will enable the testing of key epidemiological hypotheses that have been advanced regarding critical period and cumulative effects on disease risk. For some phenotypes, however, lifecourse MR may not be able to usefully contribute. This could either be due to the lack of identified genetic variants allowing meaningful separation of measures at different life stages or because these do not exist. If the IV-exposure effects are relatively constant, “standard” MR may therefore be sufficient. Awareness of this may change over time as more data becomes available. The collection of these data is also likely to be useful to improve MR overall. For example, stratifying analyses by age could be of value for testing other MR assumptions. An instrument that has very little effect on the earlier life exposure whilst influencing a later-life exposure and associating with an early-life outcome may be indicative of violations of horizontal pleiotropy, correlated pleiotropy, as well as the gene-environment equivalence (‘consistency’) assumption. In addition, lifecourse data may be used for evidence of substantial in utero effects of variants on processes suggesting developmental trajectories.

## Conclusions

There is a growing body of research focused on the development of lifecourse MR techniques and methods which are increasingly being applied to address lifecourse research questions. The possibility that genetic effects have different levels of importance in the development of an exposure at different time points should be more commonly considered for application when conducting MR investigations. The underlying assumptions for each of the methods presented in this review require careful consideration and interpretations following these analyses rely on specific condition’s which are dependent on the question being addressed, the model chosen, instruments selected and data available. We do not promote a particular strategy for conducting MR analyses in a lifecourse setting, however, we encourage practitioners to use this review to make informed decisions on how to approach a research question in this field with a solid understanding of the limitations present and how these may be affected by the aforementioned research conditions. Despite these challenges, the methodological developments and applied research being conducted using these approaches indicate the increase in opportunities becoming more available within this area.

## Supplementary Information

Below is the link to the electronic supplementary material.

Supplementary file1 (DOCX 16 KB)

Supplementary file2 (DOCX 18 KB)

Supplementary file3 (XLSX 48 KB)

Supplementary file4 (XLSX 63 KB)
