# Supplementary material for: Methodological approaches, challenges, and opportunities in the application of Mendelian randomisation to lifecourse epidemiology: A systematic literature review
Source: Eur J Epidemiol. 2023 Nov 8;39(5):501–20. doi: 10.1007/s10654-023-01032-1 (PMC7616129; doi:10.1007/s10654-023-01032-1)
Supplement: Supplementary file 1 — Supplementary file1 (DOCX 16 KB) [file 10654_2023_1032_MOESM1_ESM.docx]

**Supplementary File 1.** Search strategy used to identify lifecourse Mendelian randomisation studies

MEDLINE (PubMed)

("lifecourse"[Title/Abstract] OR "life course"[Title/Abstract] OR "time-varying"[Title/Abstract] OR "childhood adult"[Title/Abstract] OR (("childhood"[All Fields] OR "childhoods"[All Fields]) AND "later life"[Title/Abstract]) OR ("early"[All Fields] AND "later life"[Title/Abstract]) OR ("early"[All Fields] AND "life adult"[Title/Abstract]) OR (("birth s"[All Fields] OR "birthed"[All Fields] OR "birthing"[All Fields] OR "parturition"[MeSH Terms] OR "parturition"[All Fields] OR "birth"[All Fields] OR "births"[All Fields]) AND ("adult"[MeSH Terms] OR "adult"[All Fields] OR "adults"[All Fields] OR "adult s"[All Fields])) OR (("birth s"[All Fields] OR "birthed"[All Fields] OR "birthing"[All Fields] OR "parturition"[MeSH Terms] OR "parturition"[All Fields] OR "birth"[All Fields] OR "births"[All Fields]) AND "later"[All Fields] AND ("life"[MeSH Terms] OR "life"[All Fields])) OR (("gestate"[All Fields] OR "gestated"[All Fields] OR "gestates"[All Fields] OR "gestating"[All Fields] OR "gestational"[All Fields] OR "gestations"[All Fields] OR "pregnancy"[MeSH Terms] OR "pregnancy"[All Fields] OR "gestation"[All Fields]) AND ("adult"[MeSH Terms] OR "adult"[All Fields] OR "adults"[All Fields] OR "adult s"[All Fields])) OR (("gestate"[All Fields] OR "gestated"[All Fields] OR "gestates"[All Fields] OR "gestating"[All Fields] OR "gestational"[All Fields] OR "gestations"[All Fields] OR "pregnancy"[MeSH Terms] OR "pregnancy"[All Fields] OR "gestation"[All Fields]) AND "later"[All Fields] AND ("life"[MeSH Terms] OR "life"[All Fields])) OR (("uterus"[MeSH Terms] OR "uterus"[All Fields] OR "utero"[All Fields]) AND ("adult"[MeSH Terms] OR "adult"[All Fields] OR "adults"[All Fields] OR "adult s"[All Fields])) OR (("uterus"[MeSH Terms] OR "uterus"[All Fields] OR "utero"[All Fields]) AND "later"[All Fields] AND ("life"[MeSH Terms] OR "life"[All Fields])) OR (("intrauterin"[All Fields] OR "intrauterine"[All Fields]) AND ("adult"[MeSH Terms] OR "adult"[All Fields] OR "adults"[All Fields] OR "adult s"[All Fields])) OR (("intrauterin"[All Fields] OR "intrauterine"[All Fields]) AND "later"[All Fields] AND ("life"[MeSH Terms] OR "life"[All Fields])) OR (("fetale"[All Fields] OR "fetally"[All Fields] OR "fetals"[All Fields] OR "fetus"[MeSH Terms] OR "fetus"[All Fields] OR "fetal"[All Fields] OR "foetal"[All Fields]) AND ("adult"[MeSH Terms] OR "adult"[All Fields] OR "adults"[All Fields] OR "adult s"[All Fields])) OR (("fetale"[All Fields] OR "fetally"[All Fields] OR "fetals"[All Fields] OR "fetus"[MeSH Terms] OR "fetus"[All Fields] OR "fetal"[All Fields] OR "foetal"[All Fields]) AND "later"[All Fields] AND ("life"[MeSH Terms] OR "life"[All Fields])) OR (("fetale"[All Fields] OR "fetally"[All Fields] OR "fetals"[All Fields] OR "fetus"[MeSH Terms] OR "fetus"[All Fields] OR "fetal"[All Fields] OR "foetal"[All Fields]) AND ("adult"[MeSH Terms] OR "adult"[All Fields] OR "adults"[All Fields] OR "adult s"[All Fields])) OR (("fetale"[All Fields] OR "fetally"[All Fields] OR "fetals"[All Fields] OR "fetus"[MeSH Terms] OR "fetus"[All Fields] OR "fetal"[All Fields] OR "foetal"[All Fields]) AND "later"[All Fields] AND ("life"[MeSH Terms] OR "life"[All Fields])) OR (("offspring"[All Fields] OR "offspring s"[All Fields] OR "offsprings"[All Fields]) AND ("pregnancy"[MeSH Terms] OR "pregnancy"[All Fields] OR "pregnancies"[All Fields] OR "pregnancy s"[All Fields])) OR (("offspring"[All Fields] OR "offspring s"[All Fields] OR "offsprings"[All Fields]) AND ("maternally"[All Fields] OR "maternities"[All Fields] OR "maternity"[All Fields] OR "mothers"[MeSH Terms] OR "mothers"[All Fields] OR "maternal"[All Fields])) OR ("intergeneration"[All Fields] OR "intergenerational"[All Fields]) OR "two-generational"[All Fields]) AND ("mendelian randomization"[Title/Abstract] OR "mendelian randomisation"[Title/Abstract] )

- 251 results

MedRXiv

("lifecourse" OR "life course" OR "time-varying”) AND ("mendelian randomization” OR "mendelian randomisation")

- 91 results

Medline (Ovid)

((lifecourse or life course or time-varying or childhood adult or childhood later life or early later life or early life adult or birth adult or birth later life or fetal adult or fetal later life or foetal adult or intrauterine later life or intrauterine adult) and (mendelian randomization or mendelian randomisation))

- 110 results

Embase (Ovid)

((lifecourse or life course or time-varying or childhood adult or childhood later life or early later life or early life adult or birth adult or birth later life or fetal adult or fetal later life or foetal adult or intrauterine later life or intrauterine adult) and (mendelian randomization or mendelian randomisation))

- 154 results

Total: 606 results

Duplicates: 199

Remaining 407 + 3 additional records identified through other sources
