# Supplementary material for: Methodological approaches, challenges, and opportunities in the application of Mendelian randomisation to lifecourse epidemiology: A systematic literature review
Source: Eur J Epidemiol. 2023 Nov 8;39(5):501–20. doi: 10.1007/s10654-023-01032-1 (PMC7616129; doi:10.1007/s10654-023-01032-1)
Supplement: Supplementary file 2 — Supplementary file2 (DOCX 18 KB) [file 10654_2023_1032_MOESM2_ESM.docx]

**Supplementary File 2.** Inclusion and exclusion criteria

| **Inclusion Criteria** |  |
| --- | --- |
| Types of studies | Publication date: ≤ 12 June 2023 |
|  | Studies from any geographical location. |
|  | English publication language |
|  | Studies using Mendelian randomisation methods |
|  | Lifecourse epidemiology studies, defined as: 1) the effects of pre-gestation, gestation, early life, childhood, or adolescent exposures on adult outcomes; 2) the effects of adult exposures on adult outcomes when the adult exposure is related to a particular stage/phase of adulthood, such as menopause (e.g. the effects of age at menopause on cardiovascular disease), 3) the effects of repeated measures of a time-varying exposure on a later outcome. |
| Types of participants | All acceptable. |
| Types of exposure measures | Physical or social exposures measured on one or more than one occasion during gestation, childhood, adolescence, earlier or adult life or across generations as long as at least one measure pertains to a life stage before outcome is measured. |
| Types of outcome measures | Any health status or disease risk later in life, defined as a measure taken from a life stage after the exposure was measured. |

| **Exclusion Criteria** |  |
| --- | --- |
| Types of studies | Exclusively observational study designs that do not use Mendelian randomisation methods |
|  | Treatment guidelines documents, other reviews |
